# Supplementary material for: Positive and negative emotions during the COVID-19 pandemic: A longitudinal survey study of the UK population
Source: PLoS One. 2024 Feb 7;19(2):e0297214. doi: 10.1371/journal.pone.0297214 (PMC10849398; doi:10.1371/journal.pone.0297214)
Supplement: S1 File — (DOCX) [file pone.0297214.s001.docx]

# **To Zoom or not to Zoom: How has your lifestyle changed during lockdown?**

**Q1. Please indicate your response...**

| I've read the above - take me to the survey |
| --- |
| I no longer want to take part - end survey now |

*** Which statement most applies to you? (This question was added in Survey Part 4&5)**

| I am shielding |
| --- |
| I am self-isolating or quarantining |
| I am social distancing where possible |
| I am social distancing to some extent |
| None of the above |
| Other comments regarding your situation |

*** Which Tier are you in? (This question was added in Survey Part 4&5)**

| Tier 1 |
| --- |
| Tier 2 |
| Tier 3 |
| Tier 4 |
| None of the above (please specify) |

| *** On 19th July, most COVID-19 restrictions in England were lifted while citizens are urged to make responsible personal decisions. Are you going to... (This question was added in Survey Part 6)** | | |
| --- | --- | --- |
|  | Yes | No |
| Continue wearing masks in venues where it is not legally required (eg., grocery store)? |  |  |
| Continue social distancing indoors? |  |  |
| Visit crowded indoor venues, such as clubs and pubs? |  |  |
| Organize or attend parties at home? |  |  |
| Attend mass gathering events/activities, such as football and concerts? |  |  |
| Become more cautious than before July 19th due to the increasing number of cases and risky behaviour of some citizens after the Freedom Day? |  |  |
| Other (please specify) |  |  |

| *** Given the expected increase in activity post-Freedom Day, are you more or less cautious in your overall behaviour after July 19th? (This question was added in Survey Part 6)** |
| --- |
| Choices |
| Much more cautious |
| Slightly more cautious |
| The same |
| Slightly less cautious |
| Much less cautious |
| Please add any other comments surrounding changes in the way you do this activity after 19th July or which activity you stopped during the pandemics and have NOW started doing it again. |

**Q2. Email address: we will use this to invite you to complete this survey again at the specified times as mentioned above. We will not share email addresses with third parties. If you do not want to provide your email address, please choose a character name and add '@email.com' to the end. e.g. dangermouse@email.com**

## **Section 1. Activity.**

| **Q3. My typical week BEFORE 23rd March 2020 (or non-UK lockdown introduction date equivalent) involved...** | | | | | | |
| --- | --- | --- | --- | --- | --- | --- |
|  | Never | Monthly | Once per week | 2-3 times per week | Daily | More than once per day |
| Group activities with others e.g. exercise class, religious meetings |  |  |  |  |  |  |
| Spending time with family e.g. partner, children, others who you live with |  |  |  |  |  |  |
| Spending time with others e.g. extended family, friends, colleagues, others you do not live with |  |  |  |  |  |  |
| Cultural activities e.g. going to the cinema, concerts, visiting libraries, museums |  |  |  |  |  |  |
| Relaxation and taking time out e.g. meditation, doing nothing, resting |  |  |  |  |  |  |
| Getting active e.g. walking, running, going to the gym, cycling, swimming |  |  |  |  |  |  |
| Your interests e.g. surfing the internet, playing games, playing a musical instrument, writing |  |  |  |  |  |  |
| Keeping up-to-date with the world e.g. watching TV, listening to radio, reading, reading the news |  |  |  |  |  |  |
| Keeping up-to-date with social media networks e.g. Facebook, Instagram, Twitter etc. |  |  |  |  |  |  |
| In and around your home e.g. cleaning the house, food shopping, home improvements, DIY |  |  |  |  |  |  |
| Working or studying e.g. employment, studying at college or university for a qualification |  |  |  |  |  |  |
| Travelling to and from work e.g. going to a specific place to undertake a job for which you are paid |  |  |  |  |  |  |
| Helping others e.g. volunteering, freely offering to help a person, group or organisation |  |  |  |  |  |  |
| Looking after a pet e.g. walking your dog |  |  |  |  |  |  |
| Journaling e.g. handwritten, typed, art journaling, online blogging, etc (This question was added since Survey Part 3) |  |  |  |  |  |  |
| Shopping e.g. groceries, household items, clothing, non-essentials (This question was added since Survey Part 3) |  |  |  |  |  |  |

**Q4. My typical week NOW since 23rd March 2020 (the day the UK Government imposed lockdown regulations) involves…/At the moment, how often are you…**

Group activities with others e.g. group activity, exercise class, religious meetings

- Never
- Monthly
- Once per week
- 2-3 times per week
- Daily
- More than once per day

*** Have you begun new group activities or the activity you stopped during the pandemics and have NOW started doing it again?(This question was added in Survey Part 6)**

- No
- Yes, please specify

| **Q5. What has changed, if anything, about the way you do this type of activity?**   - Nothing has changed - I now do this activity online / virtually - I do not do this anymore - Instead of / in addition to my previous activity, I have taken up a new activity and this is... - I do some online, some in person (This option was added since Survey Part 4)   Please add any other comments surrounding changes in the way you do this activity.(This option was added since Survey Part 4) |
| --- |
| **Q6. At the moment, how often are you…**  Spending time with family e.g. partner, children, others who you live with   - Never - Monthly - Once per week - 2-3 times per week - Daily - More than once per day |

**Q7. What has changed, if anything, about the way you do this type of activity?**

- Nothing has changed
- I now do this activity online / virtually
- I do not do this anymore
- Instead of / in addition to my previous activity, I have taken up a new activity and this is...
- I do some online, some in person (This option was added since Survey Part 4)

Please add any other comments surrounding changes in the way you do this activity.(This option was added since Survey Part 4)

| **Q8. At the moment, how often are you...**  Spending time with others e.g. extended family, friends, colleagues, others you do not live with   - Never - Monthly - Once per week - 2-3 times per week - Daily - More than once per day |
| --- |

**Q9. What has changed, if anything, about the way you do this type of activity?**

- Nothing has changed
- I now do this activity online / virtually
- I do not do this anymore
- Instead of / in addition to my previous activity, I have taken up a new activity and this is...
- I do some online, some in person (This option was added since Survey Part 4)

Please add any other comments surrounding changes in the way you do this activity.(This option was added since Survey Part 4)

**Q10. At the moment, how often are you...**

Cultural activities e.g. going to the cinema, concerts, visiting libraries, museums

- Never
- Monthly
- Once per week
- 2-3 times per week
- Daily
- More than once per day

*** Have you begun new group activities or the activity you stopped during the pandemics and have NOW started doing it again?(This question was added in Survey Part 6)**

- No
- Yes, please specify

**Q11. What has changed, if anything, about the way you do this type of activity?**

- Nothing has changed
- I now do this activity online / virtually
- I do not do this anymore
- Instead of / in addition to my previous activity, I have taken up a new activity and this is...
- I do some online, some in person (This option was added since Survey Part 4)

Please add any other comments surrounding changes in the way you do this activity. (This option was added since Survey Part 4)

**Q12. At the moment, how often are you...**

Relaxation and taking time out e.g. meditation, doing nothing, resting

- Never
- Monthly
- Once per week
- 2-3 times per week
- Daily
- More than once per day

*** Have you begun new group activities or the activity you stopped during the pandemics and have NOW started doing it again?(This question was added in Survey Part 6)**

- No
- Yes, please specify

**Q13. What has changed, if anything, about the way you do this type of activity?**

- Nothing has changed
- I now do this activity online / virtually
- I do not do this anymore
- Instead of / in addition to my previous activity, I have taken up a new activity and this is...
- I do some online, some in person (This option was added since Survey Part 4)

Please add any other comments surrounding changes in the way you do this activity. (This option was added since Survey Part 4)

**Q14. At the moment, how often are you...**

Getting active e.g. walking, running, going to the gym, cycling, swimming

- Never
- Monthly
- Once per week
- 2-3 times per week
- Daily
- More than once per day

*** Have you begun new group activities or the activity you stopped during the pandemics and have NOW started doing it again?(This question was added in Survey Part 6)**

- No
- Yes, please specify

**Q15. What has changed, if anything, about the way you do this type of activity?**

- Nothing has changed
- I now do this activity online / virtually
- I do not do this anymore
- Instead of / in addition to my previous activity, I have taken up a new activity and this is...
- I do some online, some in person (This option was added since Survey Part 4)

Please add any other comments surrounding changes in the way you do this activity. (This option was added since Survey Part 4)

**Q16. At the moment, how often are you...**

Your interests e.g. surfing the internet, playing games, playing a musical instrument, writing

- Never
- Monthly
- Once per week
- 2-3 times per week
- Daily
- More than once per day

*** Have you begun new group activities or the activity you stopped during the pandemics and have NOW started doing it again?(This question was added in Survey Part 6)**

- No
- Yes, please specify

**Q17. What has changed, if anything, about the way you do this type of activity?**

- Nothing has changed
- I now do this activity online / virtually
- I do not do this anymore
- Instead of / in addition to my previous activity, I have taken up a new activity and this is...
- I do some online, some in person (This option was added since Survey Part 4)

Please add any other comments surrounding changes in the way you do this activity. (This option was added since Survey Part 4)

**Q18. At the moment, how often are you...**

Keeping up-to-date with the world e.g. watching TV, listening to radio, reading, reading the news

- Never
- Monthly
- Once per week
- 2-3 times per week
- Daily
- More than once per day

**Q19. What has changed, if anything, about the way you do this type of activity?**

- Nothing has changed
- I now do this activity online / virtually
- I do not do this anymore
- Instead of / in addition to my previous activity, I have taken up a new activity and this is...
- I do some online, some in person (This option was added since Survey Part 4)

Please add any other comments surrounding changes in the way you do this activity. (This option was added since Survey Part 4)

**Q20. At the moment, how often are you...**

Keeping up-to-date with social media networks e.g. Facebook, Instagram, Twitter etc.

- Never
- Monthly
- Once per week
- 2-3 times per week
- Daily
- More than once per day

**Q21. What has changed, if anything, about the way you do this type of activity?**

- Nothing has changed
- I do not do this anymore
- Instead of / in addition to my previous activity, I have taken up a new activity and this is...

**Q22. At the moment, how often are you...**

In and around your home e.g. cleaning the house, food shopping, home improvements, DIY

- Never
- Monthly
- Once per week
- 2-3 times per week
- Daily
- More than once per day

**Q23. What has changed, if anything, about the way you do this type of activity?**

- Nothing has changed
- I now do this activity online / virtually
- I do not do this anymore
- Instead of / in addition to my previous activity, I have taken up a new activity and this is...
- I do some online, some in person (This option was added since Survey Part 4)

Please add any other comments surrounding changes in the way you do this activity. (This option was added since Survey Part 4)

**Q24. At the moment, how often are you...**

Working or studying e.g. employment, studying at college or university for a qualification

- Never
- Monthly
- Once per week
- 2-3 times per week
- Daily
- More than once per day

**Q25. What has changed, if anything, about the way you do this type of activity?**

- Nothing has changed
- I now do this activity online / virtually
- I do not do this anymore
- Instead of / in addition to my previous activity, I have taken up a new activity and this is...
- I do some online, some in person (This option was added since Survey Part 4)

Please add any other comments surrounding changes in the way you do this activity. (This option was added since Survey Part 4)

**Q26. At the moment, how often are you...**

Travelling to and from work e.g. going to a specific place to undertake a job for which you are paid

- Never
- Monthly
- Once per week
- 2-3 times per week
- Daily
- More than once per day

**Q27. What has changed, if anything, about the way you do this type of activity?**

- Nothing has changed
- I now do this activity online / virtually
- I do not do this anymore
- Instead of / in addition to my previous activity, I have taken up a new activity and this is...
- I do some online, some in person (This option was added since Survey Part 4)

Please add any other comments surrounding changes in the way you do this activity. (This option was added since Survey Part 4)

**Q28. At the moment, how often are you...**

Helping others e.g. volunteering, freely offering to help a person, group or organisation

- Never
- Monthly
- Once per week
- 2-3 times per week
- Daily
- More than once per day

**Q29. What has changed, if anything, about the way you do this type of activity?**

- Nothing has changed
- I now do this activity online / virtually
- I do not do this anymore
- Instead of / in addition to my previous activity, I have taken up a new activity and this is...
- I do some online, some in person (This option was added since Survey Part 4)

Please add any other comments surrounding changes in the way you do this activity. (This option was added since Survey Part 4)

**Q30. At the moment, how often are you...**

Looking after a pet e.g. walking your dog

- Never
- Monthly
- Once per week
- 2-3 times per week
- Daily
- More than once per day

**Q31. What has changed, if anything, about the way you do this type of activity?**

- Nothing has changed
- I now do this activity online / virtually
- I do not do this anymore
- Instead of / in addition to my previous activity, I have taken up a new activity and this is...
- I do some online, some in person (This option was added since Survey Part 4)

Please add any other comments surrounding changes in the way you do this activity. (This option was added since Survey Part 4)

*** At the moment, how often are you...**

Journaling e.g. handwritten, typed, art journaling, online blogging, etc

- Never
- Monthly
- Once per week
- 2-3 times per week
- Daily
- More than once per day

*** What has changed, if anything, about the way you do this type of activity?**

- Nothing has changed
- I now do this activity online / virtually
- I do not do this anymore
- Instead of / in addition to my previous activity, I have taken up a new activity and this is...
- I do some online, some in person (This option was added since Survey Part 4)

Please add any other comments surrounding changes in the way you do this activity. (This option was added since Survey Part 4)

*** At the moment, how often are you...**

Shopping e.g. groceries, household items, clothing, non-essentials

- Never
- Monthly
- Once per week
- 2-3 times per week
- Daily
- More than once per day

*** What has changed, if anything, about the way you do this type of activity?**

- Nothing has changed
- I now do this activity online / virtually
- I do not do this anymore
- Instead of / in addition to my previous activity, I have taken up a new activity and this is...
- I do some online, some in person (This option was added since Survey Part 4)

Please add any other comments surrounding changes in the way you do this activity. (This option was added since Survey Part 4)

## **Section 2. Demographic background**

**Q41. Age**

- 18-24
- 25-34
- 34-44
- 45-54
- 55-64
- 65+
- Prefer not to say

**Q34. Gender**

- Male
- Female
- Other
- Prefer not to say

**Q35. What is your ethic group?**

- White (Welsh, English, Scottish, Northern Irish, British)
- White (Irish)
- White (Gypsy or Irish Traveller)
- White (Gypsy or Irish Traveller)
- White (other)
- White and Black Caribbean
- White and Black African
- White and Asian
- Mixed / multiple ethnic background (other)
- Indian
- Pakistani
- Bangladeshi
- Chinese
- Asian background (other)
- African
- Caribbean
- Black / African / Caribbean (other)
- Arab
- Other

**Q36. Highest level of education completed**

- Less than secondary
- Secondary up to 16 years (GCSE, O-levels etc.)
- Higher or secondary or further education (e.g. A-levels, BTEC etc.)
- College or university (e.g. BSc, BMus, professional qualification etc.)
- Post-graduate degree (e.g. MSc, MRes, PhD etc.)

**Q37. Employment status prior to the pandemic/ (Rephrased to Employment status)**

- Employed (working 0-10 hours per week)
- Employed (working 10-20 hours per week)
- Employed (working 20-40 hours per week)
- Employed (working more than 40 hours per week)
- Self-employed
- Not employed, looking for work
- Not employed, NOT looking for work
- Retired
- Not able to work

**Q38. Including yourself, how many people live in your household now?**

- I live alone
- 2
- 3
- 4
- 5 or more
- Prefer not to say

**Q39. How many of your household are under 18?**

- None
- 1
- 2
- 3 or more
- Prefer not to say

**Q40. How many of your household are 70 or over?**

- None
- 1
- 2
- 3 or more
- Prefer not to say

**Q41. Is there anyone in your household who falls into the UK Government category of 'vulnerable' (e.g. pregnant women, those who are ill or immunocompromised)?**

- None
- 1
- 2
- 3 or more
- Prefer not to say

**Q42. Do you have a garden?**

- Yes - private
- Yes - communal
- No

**Q43. Which region of the UK do you live in?**

- East of England
- East Midlands
- London
- North East
- North West
- Northern Ireland
- Scotland
- South East
- South West
- Wales
- West Midlands
- Yorkshire / Humberside
- I don't live in the UK

**Q44. We are planning to carry out phone interviews to further learn about people's experiences with lockdown during the pandemic. Would you be interested in taking part?**

- Yes - I'm happy for you to contact me via email about phone interviews
- No - please do not contact me about this

**Q45. Are you currently experiencing symptoms associated with COVID-19 (e.g. shortness of breath, high temperature, new and persistent cough)?**

- Yes
- No
- Unsure
- Prefer not to say

**Q46. Have you ever received a positive diagnosis for COVID-19 either from a healthcare professional or from a self-testing kit?**

- Yes
- No
- Prefer not to say

**Q47. Do you suspect you may have had COVID-19 but were not tested?**

- Yes
- No
- Unsure
- Prefer not to say

**Q48. The results from this survey will also feed into the development of a new and exciting lockdown journaling app. Would you like to be notified when the #MyLockdownJournal app is available to download?**

- Yes
- No

*** Have you already received a COVID-19 vaccine? (This question was added in Survey Part 4-6)**

- Yes, 1 dose
- Yes, 2 doses
- No

*** If you have not already received a COVID-19 vaccine, please state how much you agree with the following statement. (This question was added in Survey Part 4-6)**

|  | Strongly Agree | Agree | Neither agree nor disagree | Disagree | Strongly disagree |
| --- | --- | --- | --- | --- | --- |
| I would be willing to take COVID-19 vaccine only if it is mandantory for my work |  |  |  |  |  |
| I would be willing to take COVID-19 vaccine only if it is mandantory for my travel |  |  |  |  |  |
| I would not be willing to take any COVID-19 vaccine no matter what happens |  |  |  |  |  |

**Q41. If you remain sceptical about taking a UK Gov approved vaccine, please select your reasons why or choose the 'Other' option and tell us more. (This question was added in Survey Part 4-5)**

- I am worried about potential side effects of a vaccine
- My friends or family may not approve
- I would want to wait for more information
- I have a fear of needles
- I do not believe the COVID-19 to be a real pandemic
- I believe I am immune to COVID-19
- I would only take it if it is mandatory for working or travelling
- I do not mind being infected with a vaccine
- Other (please specify)

## **Section 3. Mental health status**

*** We would now like you to think about how you have felt over the PAST FEW DAYS. Please read each item below and rate your response by selecting a point on the scale. Try to be honest and don't worry, there are no right or wrong answers.**

|  | Not at all | A little | Moderately | Quite a bit | All the time |
| --- | --- | --- | --- | --- | --- |
| Interested |  |  |  |  |  |
| Distressed |  |  |  |  |  |
| Excited |  |  |  |  |  |
| Upset |  |  |  |  |  |
| Strong |  |  |  |  |  |
| Guilty |  |  |  |  |  |
| Scared |  |  |  |  |  |
| Hostile |  |  |  |  |  |
| Enthusiastic |  |  |  |  |  |
| Proud |  |  |  |  |  |
| Irritable |  |  |  |  |  |
| Alert |  |  |  |  |  |
| Ashamed |  |  |  |  |  |
| Inspired |  |  |  |  |  |
| Nervous |  |  |  |  |  |
| Determined |  |  |  |  |  |
| Attentive |  |  |  |  |  |
| Jittery |  |  |  |  |  |
| Active |  |  |  |  |  |
| Afraid |  |  |  |  |  |
