# Supplementary material for: Positive and negative emotions during the COVID-19 pandemic: A longitudinal survey study of the UK population
Source: PLoS One. 2024 Feb 7;19(2):e0297214. doi: 10.1371/journal.pone.0297214 (PMC10849398; doi:10.1371/journal.pone.0297214)
Supplement: S2 File — (DOCX) [file pone.0297214.s002.docx]

**Appendix C. Reliability assessment**

**Tables 1. Cronbach alpha if the item is deleted, corrected item-total score correlations, and correlations between items (PANAS-Positive)**

|  | **Cronbach alpha if item deleted** | **Corrected item-total correlation** | **Correlations between items** | | | | | | | | | |
| --- | --- | --- | --- | --- | --- | --- | --- | --- | --- | --- | --- | --- |
|  |  |  | **Interested** | **Excited** | **Strong** | **Enthusiastic** | **Proud** | **Alert** | **Inspired** | **Determined** | **Attentive** | **Active** |
| Interested | 0.900 | 0.695 | 1.000 | 0.449 | 0.526 | 0.596 | 0.383 | 0.479 | 0.500 | 0.502 | 0.563 | 0.446 |
| Excited | 0.903 | 0.644 |  | 1.000 | 0.445 | 0.665 | 0.473 | 0.320 | 0.561 | 0.429 | 0.397 | 0.339 |
| Strong | 0.898 | 0.733 |  |  | 1.000 | 0.578 | 0.482 | 0.489 | 0.504 | 0.622 | 0.549 | 0.477 |
| Enthusiastic | 0.892 | 0.826 |  |  |  | 1.000 | 0.566 | 0.477 | 0.676 | 0.586 | 0.556 | 0.475 |
| Proud | 0.904 | 0.638 |  |  |  |  | 1.000 | 0.369 | 0.537 | 0.521 | 0.429 | 0.340 |
| Alert | 0.903 | 0.654 |  |  |  |  |  | 1.000 | 0.425 | 0.520 | 0.633 | 0.456 |
| Inspired | 0.897 | 0.747 |  |  |  |  |  |  | 1.000 | 0.592 | 0.529 | 0.406 |
| Determined | 0.895 | 0.771 |  |  |  |  |  |  |  | 1.000 | 0.637 | 0.468 |
| Attentive | 0.897 | 0.759 |  |  |  |  |  |  |  |  | 1.000 | 0.490 |
| Active | 0.905 | 0.601 |  |  |  |  |  |  |  |  |  | 1.000 |

All correlations were statistically significant at *p* < .01 (two-tailed);

**Tables 2. Cronbach alpha if the item is deleted, corrected item-total score correlations, and correlations between items (PANAS-Negative)**

|  | **Cronbach alpha if item deleted** | **Corrected item-total correlation** | **Correlations between items** | | | | | | | | | |
| --- | --- | --- | --- | --- | --- | --- | --- | --- | --- | --- | --- | --- |
|  |  |  | **Distressed** | **Upset** | **Guilty** | **Scared** | **Hostile** | **Irritable** | **Ashamed** | **Nervous** | **Jittery** | **Afraid** |
| Distressed | 0.878 | 0.764 | 1.000 | 0.749 | 0.327 | 0.547 | 0.422 | 0.500 | 0.319 | 0.551 | 0.559 | 0.585 |
| Upset | 0.878 | 0.773 |  | 1.000 | 0.349 | 0.538 | 0.458 | 0.534 | 0.324 | 0.544 | 0.542 | 0.575 |
| Guilty | 0.896 | 0.498 |  |  | 1.000 | 0.343 | 0.237 | 0.304 | 0.520 | 0.335 | 0.316 | 0.314 |
| Scared | 0.879 | 0.759 |  |  |  | 1.000 | 0.314 | 0.360 | 0.298 | 0.676 | 0.581 | 0.809 |
| Hostile | 0.893 | 0.534 |  |  |  |  | 1.000 | 0.539 | 0.293 | 0.317 | 0.356 | 0.339 |
| Irritable | 0.888 | 0.631 |  |  |  |  |  | 1.000 | 0.301 | 0.438 | 0.473 | 0.392 |
| Ashamed | 0.895 | 0.493 |  |  |  |  |  |  | 1.000 | 0.326 | 0.317 | 0.319 |
| Nervous | 0.877 | 0.776 |  |  |  |  |  |  |  | 1.000 | 0.711 | 0.720 |
| Jittery | 0.879 | 0.749 |  |  |  |  |  |  |  |  | 1.000 | 0.645 |
| Afraid | 0.876 | 0.801 |  |  |  |  |  |  |  |  |  | 1.000 |

All correlations were statistically significant at *p* < .01 (two-tailed)


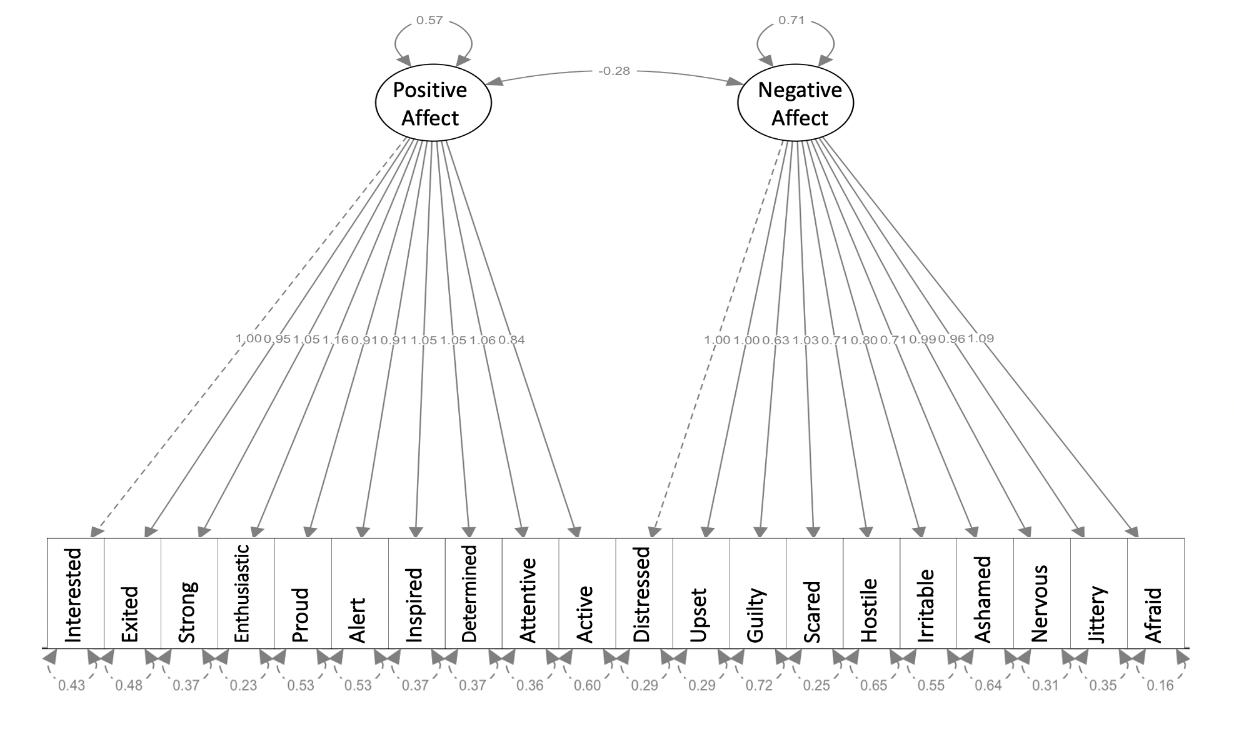


**Figure 3. Confirmatory factor analysis (CFA) model. Note. Rectangles are measured variables, the large ovals are the latent construct, and ellipses are residual variances. Factor loadings are standardized**

Interpretation: All factor loadings were statistically significant (p < .001). The covariance (-0.284) between the positive and negative affect is statistically significant (p<0.001), and the correlation is -0.448. With regard to the first factor (positive affect), all standardised loadings were in the range from .84 to 1.16. Regarding the second factor (negative affect), standardised loadings were large, with a minimum of 0.63 and a maximum of 1.09.

**Table 4. Model fit indices**

| Measures | Result | Threshold |
| --- | --- | --- |
| χ2(169) | 13579.047 | - |
| P-value for the model | < .001 | - |
| CFI | .940 | Great: >.95  Acceptable: >.90 |
| TLI | .932 | Great: >.95  Acceptable: >.90 |
| SRMR | .072 | <.09 |
| RMSEA | 0.099 CI [.098–.101] | Good: <.05  Moderate: .05-.10  Bad:>.10 |
| *Note*. CI = confidence interval; CFI = Comparative Fit Index; TLI = Tucker–Lewis index; SRMR = Standardized Root Mean Square Residual; RMSEA = Root Mean Square Error of Approximation | | |

Interpretation:

The value (Comparative Fit Index) CFI, Tucker-Lewis Index (TLI) are higher than .90, which indicates a good fit, while the Root Mean Square Error of Approximation (RMSEA) was a little higher than expected (>0.08). However, taking into account that the parameter estimates were all statistically significant and very large, we can conclude that the model has an adequate fit.
