# Supplementary material for: Positive and negative emotions during the COVID-19 pandemic: A longitudinal survey study of the UK population
Source: PLoS One. 2024 Feb 7;19(2):e0297214. doi: 10.1371/journal.pone.0297214 (PMC10849398; doi:10.1371/journal.pone.0297214)

**S3 File. Histogram and Q-Q plot for checking data distribution**

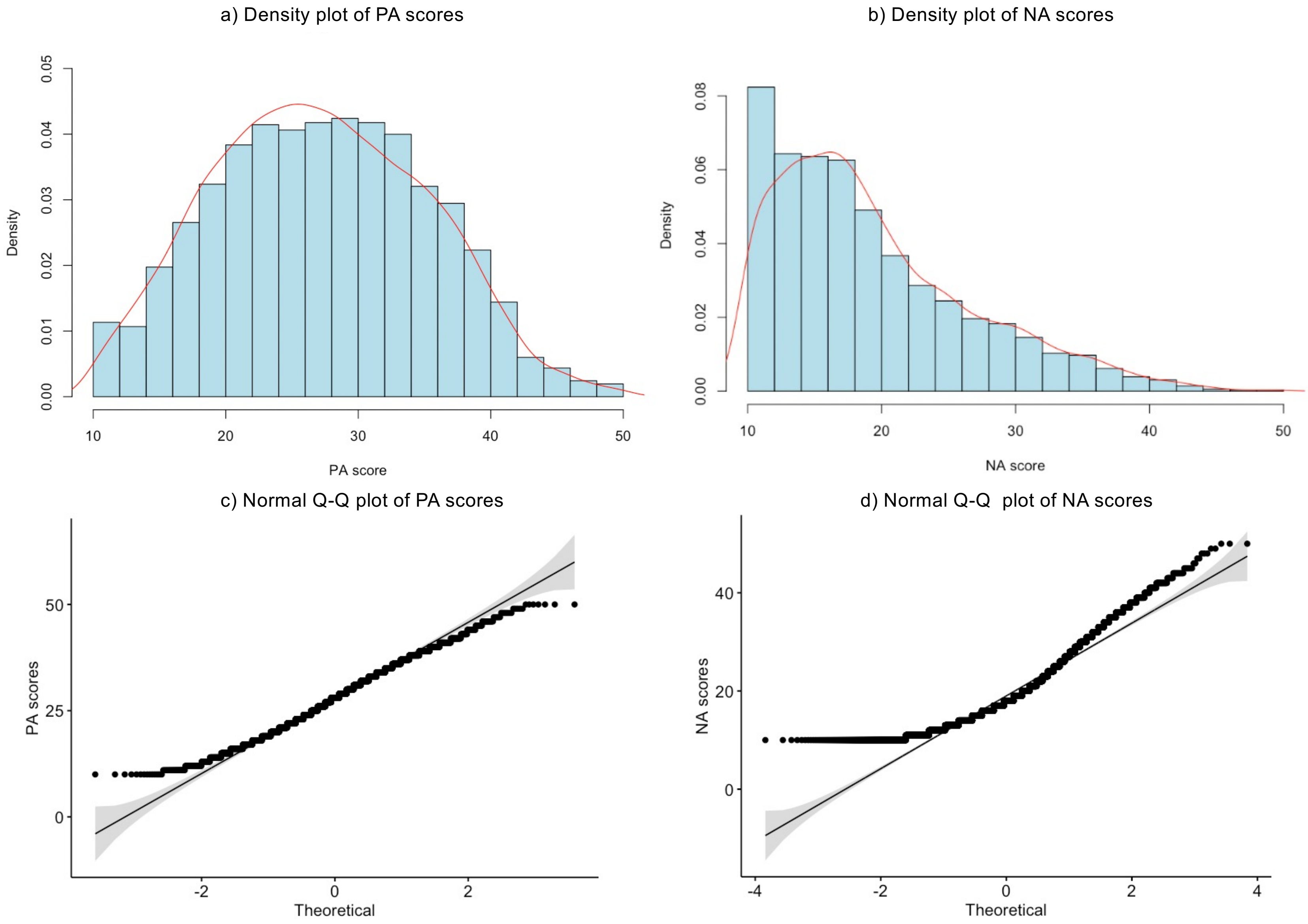

Supplement: S3 File — (PDF) [file pone.0297214.s003.pdf]
