# Supplementary material for: Positive and negative emotions during the COVID-19 pandemic: A longitudinal survey study of the UK population
Source: PLoS One. 2024 Feb 7;19(2):e0297214. doi: 10.1371/journal.pone.0297214 (PMC10849398; doi:10.1371/journal.pone.0297214)
Supplement: Attachment — Submitted filename: Covid_UK_Plos1.docx [file pone.0297214.s007.docx]

Summary

The paper studies the changes of emotional states of people throughout different stages and policies of the COVID pandemic. The paper uses PANAS emotional state measures of positive and emotional states of 4222 people (and 167 observed repeatedly) in UK between March 2020 and October 2021. Two separate study designs are used. The first one is the cross-sectional study which looks at the difference of the emotional states across various demographic groups. The second one is a prospective cohort study that explores the changes throughout the stages of the pandemic. The authors find significant differences for the stages 2, 5, and 6, which are the stages with relaxed COVID measures and Freedom Day. There are also significant differences across the demographic groups, finding older, retired and female population groups to be the most positive.

The major contribution claimed by the authors is the fact that this study is longitudinal while most previous studies are based on cross-sectional analysis. However, the repeated sample is fairly small (167 individuals). Another contribution, as the authors point it out, is the fact that this is the first paper to use PANAS in UK throughout the whole pandemic. The most significant shortcoming is non-representative sample used in the paper. While all the groups are there, the sample mainly consists of female older and educated population from Southern England.

Suggestions
The paper is generally well written and covers an interesting topic which is current and relevant. My minor comments are below:

1. Figure 1 is hard to read, especially the small text below the x-line. Figure 5 – the numbers are unreadable. Figure 4: the negative score graph does not line up exactly with the stages like the positive score graph. It creates confusion for the reader as it appears that they are done at different stages.
2. The authors state on page 4 that “most studies are cross-sectional surveys”. Does this mean that there are some studies that are not cross-sectional surveys? How does this paper compare to those studies then? It would be nice to see a more thorough discussion on that in the introduction.
3. P12: When interpreting, “people who live with five or more households have the highest PA and lowest NA scores, but the difference Is insignificant…” This doesn’t really make sense. So are they highest or are they the same then? If it is insignificant, then there is no difference statistically. The authors should state the highest/lowest significant group instead.
4. There are a few typos scattered throughout the paper. Careful proofreading would solve the issue.
