# Supplementary material for: Positive and negative emotions during the COVID-19 pandemic: A longitudinal survey study of the UK population
Source: PLoS One. 2024 Feb 7;19(2):e0297214. doi: 10.1371/journal.pone.0297214 (PMC10849398; doi:10.1371/journal.pone.0297214)
Supplement: Attachment — Submitted filename: Response to Reviewers.docx [file pone.0297214.s008.docx]

Dear Prof M Niaz Asadullah,

Dear Reviewers,

Thank you for giving us the opportunity to submit a revised version of our manuscript titled ‘*Positive and Negative emotions during the COVID-19 Pandemic: A longitudinal survey study of the UK Population*’ to PLOS ONE to be considered for publication as *Research Article.*

We appreciate the effort and time the editor and the reviewers have dedicated in providing valuable feedback on the manuscript and we are grateful for all comments. We have incorporated adaptions that reflect upon the suggestions and we have been able to address all the issues raised in the feedback. The remarks have been immensely helpful and we are convinced that the manuscript improved substantially.

We include the comments provided by the reviewer and the editor immediately in the tables below and corresponds to our response and revisions. Please note that all the page and line numbers cited in this document are corresponded to the file “Manuscript”, thanks!

We are very much looking forward to your decision and greatly appreciate your attention and valuable time.

Yours sincerely,

Lan Li

On behalf of the co-authors.

| **Response to Editor** | | |
| --- | --- | --- |
| **No** | **Comment** | **Response** |
| 1 | There is a sizable literature on mental health (MH) during COVID times which present causal evidence on exposure to pandemic induced lockdown and mental heath exploiting spatial variation in lockdown timing. The manuscript needs to acknowledge this - please update your literature review accordingly - and offer some discussion of how your approach compares and contrasts with other key studies on the topic. | - Thank you for the comments. We have added the reviews of the current longitudinal studies and updated the discussion on our approaches compared to previous studies. Please see Line 62-74 in Page 4-5. |
| 2 | Following from the above comment and taking note of concerns of selection bias highlighted by Referee-1, I expect a more carefully written version in the next round. Since the manuscript claims to offer "creative exploratory analysis and statistical tests" and in conclusion "we performed a longitudinal survey study", please clarify how you achieved this from a methodological point of view that is consistent with the literature  (including a discussion of selection  bias issues) - else, add more to rigor to your empirical analysis during the revision. I say this b/c the dataset is very rich - PANAS longitudinal survey from the UK (March 2020 to July 2021). Yet the analysis is only compares MH (+ve and -ve emotions) across different phases of lockdown. As such, the dynamic nature of the data is not fully exploited. | - Thank you for the comments. We have addressed the comments provided by the reviewers and proofread the paper carefully. The wording and phrases used in this version were improved to describe what we have achieved in this study. The creativity was achieved using various study designs and ways of data visualisation. The longitudinal study was achieved by multiple surveys launched at different time points and comparing PANAS scores between phases. The rationale for selecting the sampling method and the potential selection bias were stressed in the method, discussion & conclusion section. Please see Line 105-106, Line 325-326, and Line 352-353, respectively.  - Thank you for reassuring us that we had collected a rich dataset. We might have to clarify that this is a part of our large 3-year project “To Zoom or Not to Zoom”, as we described in our methodology section (Line 98-101, page 6). Using the quantitative and qualitative data collected throughout the pandemic, four publications will be out to present the impacts on different aspects. One of those focusing on the lifestyle change impacted by the policy was already published in PLOS ONE; please see reference 26. This is the second paper in which we aim to look for the changing of positive and negative emotions affected by the pandemic. A further paper will connect the two and explore the relationship between the activity and emotions among a certain group using mixed methods. Therefore, we acknowledge that the data provided rich information about the impact of the pandemic on the UK population; this paper aims only to look at the emotional side. As we have multiple phases of data, we attempted to deliver a clear message to our readers without overwhelming them. Instead, the dynamic nature of the data will be present in multiple publications exploring different topics, which will be better to meet the variety of readers’ interests. |
| 3 | Figures 4 and 5 are not acceptable in terms of quality and format. Please use "actual dates" or "text label" to clearly identify each phase on the x-axis instead of numbers 1-7 (you already have the name description of each phase in Fig 1). Otherwise it's difficult for readers to remember which number corresponds to which phase. | Thanks for your suggestion. We have changed the x-axis of the two graphs to text labels and added timeline descriptions into legends. We believe now it is much easy to read. Please see the updated figures. |
| 4 | In Figs 4 & 5, the legends used are not standard and not aligned with terms used in the main text. What do you mean by "Original positive score"? Either say "unadjusted score (positive)" or "raw score (positive)" | Thanks for pointing this out. To ensure consistency, we have changed the legends of Figure 4&5 (Figure 3&4 in the latest version) and double-checked the terms used in the main text. We also move the notes from the caption to the figure to improve the readability. Please see the updated figures. |
| 5 | Fig 2 looks strange. Consider adding corresponding mean values etc and add notes at the bottom to explain what the content stands for. The title is also very confusing "Positive and negative affect states measured by PANAS" b/c there is nothing on "measurement scale" in Fig 2. Please address similar concerns for all other Tables and Figs. | Thanks for this comment. Figure 2 was designed to give the readers some ideas about what is contained in PANAS scores. We do agree that it looks strange and redundant. Thus, we have removed it in the latest version as all the information could be found in the main body instead (Line 122-125, page 7). |

**Response to Editor**

| **No** | **Comment** | **Response** |
| --- | --- | --- |
| 1. | Figure 1 is hard to read, especially the small text below the x-line. Figure 5 – the numbers are unreadable. Figure 4: the negative score graph does not line up exactly with the stages like the positive score graph. It creates confusion for the reader as it appears that they are done at different stages. | Thank you for the comments.  - The fonts of the small texts in Figure 1 were increased; if it is still hard to read, we could transform it into a portrait when publishing - we would consult the typesetters for details.  - We have increased the size of the numbers in Figure 5 (see Figure 4 in the latest version);  - We have adjusted figure 4 to line up the bars (see Figure 3 in the latest version). |
| 2. | The authors state on page 4 that “most studies are cross-sectional surveys”. Does this mean that there are some studies that are not cross-sectional surveys? How does this paper compare to those studies then? It would be nice to see a more thorough discussion on that in the introduction. | Thank you, we have added the reviews on the longitudinal studies into the introduction section. Please see line 62-74, page 5. |
| 3. | P12: When interpreting, “people who live with five or more households have the highest PA and lowest NA scores, but the difference Is insignificant…” This doesn’t really make sense. So are they highest or are they the same then? If it is insignificant, then there is no difference statistically. The authors should state the highest/lowest significant group instead. | Thanks for the comments. We have revised the wording of this interpretation. Please find the updates in line 216-218, page 12. |
| 4. | There are a few typos scattered throughout the paper. Careful proofreading would solve the issue. | Thank you. We have checked and proofread the whole paper and corrected the typos. |

**Response to Reviewer #1**

| **No** | **Comment** | **Response** |
| --- | --- | --- |
| 1 | This is an interesting study taking advantage of a panel of six rounds where a sample of respondents participated in all six rounds. Such balance panels are typically hard to find, and it is commendable that the authors could take advantage of this aspect of the survey. My main concern is the sample size and nature of selection. Both of these issues will have implications for how generalizable the findings are at the national or “societal” level. | Thank you for your reassurance. We acknowledge the limitation of sample size (especially the retrospective cohort) and the selection bias; we have adjusted our wording carefully throughout the paper. We also stressed the potential selection bias in our method, discussion & conclusion section-please see Line 105-106, Line 325-326, and Line 352-353, respectively. |
| 2 | There are two types of selection issues here. One is the recruitment of the respondents was done through social media advertisement along with few other channels (p.6, lines 99-102). Hence, the people who responded may not be representative of the adults at the national level. While the time trend for the respondents in the balanced panel can be useful, any inference drawn using 167 respondents worries me. Few suggestions:  (a)How about reporting the average for the entire sample by rounds? Didn’t they take same PANAS tests? It seems so from Table 1. So, the summary stats based on repeated cross-sectional data will also be very useful as they may have more statistical information. Another benefit of showing the results from the balanced panel (N = 167) and overall sample (which varied by rounds) is that it will show the robustness of the findings and allow how representative the restricted sample is. (b) Should we consider deriving some sample weights using population wide data? Obviously, people who self-selected to take part in the survey and further to participate in all six rounds may not be similar to the population. But sample weights can perhaps partially correct the biases. | Thank you for your constructive comments. These are really helpful for us. As mentioned above, we acknowledge the potential selection bias and the limited generalizability. As per the suggestions, we would like to clarify our current approaches.  Re suggestion 1), we have reported the average of the entire sample by rounds; please see table 2. We presented the mean, SD, min and max of both positive and negative scores for each phase, based on the cross-section survey data collected in each round. We do agree the summary stats are useful to inform the reader about the general mental health status in each phase. For the cohort data, as you pointed out, we only have a small sample (N=167); we thus only aimed to present the individual changes over time using Alluvial plots using this bit data.  Re suggestion 2), it is a good point and helpful suggestion. We might have to clarify that we have already used weights in the population-wide data. As shown in figure 4, we presented the raw and adjusted average after adjusting the demographic covariates to correct the selective bias.  Nevertheless, thank you for your helpful suggestions and for pointing these issues out; we have improved our wording in the methodology and limitation section to clarify our approaches and the potential bias, as mentioned above. |
| 3 | The authors have been able to show that the changes in moods (positive and negative) coincide with the mobility restrictions. Authors may want to present the trends superimposed on a chart which shows the degree of restrictions over time. This will be a very useful visual aide to understand the association between COVID restrictions and mood of people sampled. | Thank you, we have considered this suggestion carefully. We agree that combing the two in one chart would be interesting to visualise the relationship directly. However, we finally decided not to do that after a long internal discussion among the co-authors – the main reason is that it might overwhelm the readers as the current graphs already contained much information. Further, this information was provided through texts and separated into different charts. It will be repeated if we do so. Therefore, instead, we added a legend box to reiterate each phase's time frame so that readers could easily read and understand the graphs. Please see the updated figures 2-4. |
| 4 | The authors claim about sample size to be large (p.16, lines 303-304). Should a sample size of 167 be considered large? | Thank you for pointing this out. We have rephrased this in the latest version. Please see line 317 to 319, page 16. |
| 5 | In terms of analyses, the authors have some covariates which are systematically correlated with the PANAS score. Authors may consider taking different respondent features (gender, age, etc.) and interact with round or the strictness of lockdown. The coefficients for the interaction terms may allow testing how different features are associated with the round specific mood outcomes. | Thank you for your suggestion. That’s an excellent point. We did consider interacting the demographics with the phases (round of strictness) when we performed the data analysis. However, we finally dropped it out due to the following reasons: 1) the results of the statistical tests would have less power in each phase due to reduced sample size – most of the results were insignificant; 2) it might overwhelm the readers by providing so many charts or texts to explain the difference between phases, but it couldn’t add much value to the main message that we would like to deliver. Meanwhile, as this paper sought to know the impact of the pandemic on emotional status, it would be out of scope if we went deep into the demographic differences. Instead, we provided figure 4, which breaks and compares the PANASs scores among phases after adjusting the demographic differences. However, we do agree it would be interesting to test how different demographics are associated with the round-specific mood outcomes; it could be added to further studies that focus on the demographics. |
| 6 | Associations of moods with some of the features need some elaboration. Why did women report better mental health outcomes compared to men? Is this typical in the literature? In general, the authors to discuss their findings in the context of what other studies have found so far. | Thank you for pointing this out. It is really helpful. We have added these to our discussion sections. Please see line 270-271, 274-283, page 14-15. |

**Response to Reviewer #2**

| **No** | **Comment** | **Response** |
| --- | --- | --- |
| 1 | The manuscript is well written except a couple of typos scattered throughout text. The paper uses PANAS emotional state measures of positive and emotional states of 4222 people (and 167 observed repeatedly) in UK between March 2020 and October 2021. Two separate study designs are used. The first one is the cross-sectional study which looks at the difference of the emotional states across various demographic groups. The second one is a prospective cohort study that explores the changes throughout the stages of the pandemic. | Thank you for the comment. We have proofread the whole paper and corrected the typos. |
| 2 | All the tables for the analysis are included, though some need more sharpness. | Thank you for this comment. We have improve the figure quality and sharpness. Please see the updated figure 1-4. |

**Response to Journal Requirements**

| **No** | **Comment** | **Response** |
| --- | --- | --- |
| 1 | Please ensure that your manuscript meets PLOS ONE's style requirements, including those for file naming. The PLOS ONE style templates can be found at https://journals.plos.org/plosone/s/file?id=wjVg/  PLOSOne_formatting_sample_main_body.pdf and  https://journals.plos.org/plosone/s/file?id=ba62/  PLOSOne_formatting_sample_title_authors_affiliations.pdf | Thank you for the comment. We have checked that the manuscript meets all style requirements. We also meet the requirements for file naming. |
