# Supplementary material for: Positive and negative emotions during the COVID-19 pandemic: A longitudinal survey study of the UK population
Source: PLoS One. 2024 Feb 7;19(2):e0297214. doi: 10.1371/journal.pone.0297214 (PMC10849398; doi:10.1371/journal.pone.0297214)
Supplement: Attachment — Submitted filename: Response to Reviewers.docx [file pone.0297214.s009.docx]

Dear Prof Guglielmo Campus,

Dear Reviewers,

Thank you for giving us the opportunity to submit a revised version of our manuscript titled ‘*Positive and Negative emotions during the COVID-19 Pandemic: A longitudinal survey study of the UK Population*’ to PLOS ONE to be considered for publication as *Research Article.*

We would like to express our gratitude for your time and effort in providing valuable feedback on our manuscript. We have carefully considered all comments and have made significant revisions based on the suggestions provided. We believe that the manuscript has improved substantially as a result of your feedback.

In order to make it easier to follow the changes we have made, we have included the comments provided by the reviewers in tables below, along with our responses and revisions. Please note that all page and line numbers cited in this document correspond to the file "Manuscript".

Once again, we thank you for your valuable feedback and hope that the revised manuscript meets your expectations.

We are very much looking forward to your decision and greatly appreciate your attention and valuable time.

Yours sincerely,

Lan Li

On behalf of the co-authors.

| **Response to Reviewer #3** | | |
| --- | --- | --- |
| **No** | **Comment** | **Response** |
| 1 | Thank you for your manuscript. This is the first time I have seen the paper and have not been able to see your previous response to reviewers, hence my comments take the manuscript without prior knowledge of reviews and on face value.  You present a clear introduction and rationale as to why this study may be needed considering there is limited data assessing changes in the pandemic and individuals responses to it over time. My initial impression is that the discussion could be expanded to go into more depth and engage with more literature. There are some key limitations not mentioned that should be added.  Lastly I believe there should be more concrete implications added to the manuscript to answer the 'so what' question, i.e. describing why your findings are useful and what policies, practice or research it can inform in the future. I add more detail on these issues below. I believe if these are addressed, the work will provide an interesting piece of evidence in the COVID literature in the UK. | Thank you for your valuable feedback. We appreciate your suggestions and have taken them into serious consideration. We have made several changes to the manuscript to address your concerns.  Regarding the discussion section, we have expanded it to provide a more in-depth analysis of the literature. We have included additional references and engaged more deeply with the existing research to ensure that our discussion is more comprehensive.  We have also added a section on the limitations of our study. We agree that this is an important aspect that should not be overlooked. We have carefully considered the limitations of our study and have added them to the manuscript.  Lastly, we have added more concrete implications to our findings. We believe that this is an important aspect of the manuscript, and we have taken your suggestion to heart. By addressing the "so what" question, we hope to provide more context and relevance to our study's results. |
| 2 | Line 94 – typo – should this be 'longitudinally'? | Thank you for the comments. We have corrected the typo. |
| 3 | Line 279 – why might measuring emotions, rather than psychological conditions have different presentation in the data? You state this may be the reason for finding difference, but then do not explain why this might cause a difference - e.g. I assume it is because emotions can flux more readily that probable conditions - but citing supporting research and explaining further would be useful. | Thanks for your suggestion. We have expanded this in our discussion. Please see Page 16 Line 298-302. |
|  | - Further discussion of more context of phases would be useful e.g. In the first phases there were community responses in support and action – neighbourhood and NHS volunteering, clapping emergency support workers (aka we are all in this together - however some of this community spirit may have dissipated at later restricted phases? | Thank you for this suggestion. We have added this into our discussion. Please see Page 17-18 Line 334-344. |
|  | - There is a huge amount of evidence collected overtime from the UCL Social Study, but this does not seem to be included or discussed in the discussion - it might benefit from including some of their findings in the context of your study. Additionally what international studies and you cite that have explored longitudinal data and how does this relate to your findings? | Thank you for this suggestion. We have added this into our discussion. Please see Page 15 Line 286-291 and Page 17 Line 284-344. However, as most of the studies (including UCL Social Study) only covered the first lockdown or early months of the pandemic, we can only discuss them in limited time frame. Nonetheless, they are valuable evidences for exploring demographic factors and emotional differences between Phase 1 and Phase 2. |
| 4 | Line 321 –you state the geographical limitation but don't explain how this might bias results i.e. may not take into account other areas with higher levels of social deprivation, large northern cities etc. but why limitation – | Thanks for pointing this out. Our sample is limited to participants in the UK, and while we have taken measures to ensure a diverse sample, we acknowledge that our findings may not generalize to other populations or regions. Additionally, as you mentioned, our study may not fully capture the experiences of individuals in areas with higher levels of social deprivation or in large northern cities. We have explained it to address this concern. Please see Page 19 Line 368-371. |
| 5 | Line 343 – do you mean 'cross-sectional' ? | Thanks for this comment. We have corrected the typo. |
| 6 | Limitations –you make no mention of method of recruitment as being a limitation and possible source of bias – e.g. convenience sampling through social media online. It would be good to add how this might bias who took part in your study. e.g. possible that those who had experienced more problems would be more attracted to take part - it is known in other psychological studies that those with mental health problems are more likely to take part in studies that brand themselves as mental health studies and hence may bias average distress reported in those studies. Additionally by recruiting online and through social media, may bias which populations engage with facebook (for example) and you may not have reached younger or older generations - some noting or discussion of this would be useful. | Thanks for this comment. We have added this into our limitation. Please see Page 19 Line 374-383. |
| 7 | Implications - It would be useful to discuss what implications these findings have and how/who they might be useful for - e.g. government policy? healthcare practice? future research. Currently the 'so what' question isn't answered fully enough to make these results feel tangible - hence some more discussion of tangible implications would be good. | Thank you for the suggestion. We agree that discussing the implications of our findings is crucial in providing a meaningful context for our study. Based on our results, we have added a paragraph into our discussion. Please see Page 18 Line 348-357. |

**Response to Reviewer #4**

| **No.** | **Comment** | **Response** |
| --- | --- | --- |
| 1 | This is manuscript presents an interesting exploratory analysis of changes in positive affect in a longitudinal cohort. The sample available for analysis is relatively small (n=167) and selection bias is a limitation to this study. However, the work does highlight the need to investigate changes in positive as well and negative affect in crisis situations as both dimensions are important to well-being. The PANAS also taps into fluctuation in mood states rather than assessing symptoms of psychological distress. As such, the study raises important questions for consideration and further investigation in developing pandemic response plans and policies.  I have read the previous reviewers' comments and author responses and can see that the manuscript has been significantly improved since the last version. | Thank you for your feedback and for taking the time to read through the previous reviews and our responses. We appreciate your acknowledgement of the improvements made to the manuscript. We agree that the sample size is a limitation to our study, and we have emphasized this in the revised our manuscript accordingly. We also appreciate your recognition of the importance of investigating changes in positive affect during crisis situations and the potential implications for developing pandemic response plans and policies.  Thank you for your thoughtful comments. |
| **Abstract** | | |
| 2 | • Consider rephrasing ‘paralysed the world’ | Thank you, we have rephrase the sentence. |
| 3 | • Ensure it is clear that it is positive affect sub-scale scores that are referred to in the results in this section, with higher scores indicating more positive affect. | Thank you, we have clarified this in our abstract. |
| **Methods** | | |
| 4 | • Page 6, lines 107-112. The sampling approach is a little unusual, with a second wave of recruitment taking place in October and December 2020. As such, these participants would not have completed the earlier surveys and would not have the full five data points available. There is also information about optional lifestyle questionnaires. Was the PANAS part of the optional questionnaire set or the core survey? There is some further information on selection of the sample in the ‘data cleaning’ section. This would be clearer and easier to follow in a single section, with a table or flow diagram to show exactly what data is available at each time point for cross-sectional analysis, and from all time points for longitudinal analysis. | Thanks for the comments.  Regarding the recruitment: We added a second wave of recruitment to maximize the sample size while operating within limited resources and a tight timeframe. Therefore, we utilized two study designs for our analysis. The total participants from both phases were included in the cross-sectional analysis, whereas the repeated cohort was analysed separately in the prospective study design. As a result, only 167 participants were included in the latter analysis. The PANAS questions were included in the core survey as described in the manuscript.  We have added the table detailing the sample size for each phase. Please see Table 1. We hope this clarification improves the clarity of the study. |
| 5 | • Analysis and results section mention the NA scale, but this is not covered in the abstract, introduction, or discussion. | Thank you for your comment. We have improve the interpretation in each section. |
| **Results** | | |
| 6 | • P10, Line 187, as noted in the methods it is unclear what the 8073 records relate to – is this complete PANAS data available for analysis, and for how many unique individuals at each time point – or were these pooled for analysis? | Thank you for the question. As we stated, the 8073 records were the total number of records used for analysis after the data cleaning. We have added a table in methodology section as mentioned above. We believe it is much clearer now. |
| 7 | • The demographic profile of the cohort indicates that well-educated middle and older-age females are considerably over-represented in the sample. This sampling issues are common with online surveys, but it is worth considering this during interpretation – particularly as there is ample evidence that mental health was more adversely affected in demographic groups where there were pre-existing health equalities which were exacerbated during the pandemic. | Thank you for your feedback regarding the potential sampling bias in our study. We have carefully considered your comments and have added sentences in the sample description section. Please see Page 15 Line 269-279. We have also revised our limitation section for clarifying the potential bias. Please see Page 19-20 Line 359-394. |
| 8 | • Table 2 of the results does indicate numbers of data points available for analysis at each time point, and as noted above it would be good to see this come in earlier in the paper so that this is really clear. | Thank you for your feedback. We have addressed this issue by adding the information on the number of data points available for analysis in each phase in the methodology section of the paper. Please see table 1. We hope this clarification improves the clarity of the study. |
| 9 | • Given the extent of change in participants recruited and exiting the study at different time points, it would be useful to assess the potential for selective drop-out in terms of mental health – especially given the apparent improvement in positive affect during later stages in the study. I.e. were people with higher initial PA and lower NA more likely to be lost to follow-up? | Thank you for the suggestion. We completely understand the importance of assessing the potential for selective drop-out in terms of mental health. However, we must agree that it is difficult to perform this analysis due to the limited number of responses provided by the participants. While we acknowledge that there might be a link between the drop-out rate and mental health, other factors could also influence their willingness to complete the survey, such as lack of time or interest. Nevertheless, we appreciate this valuable insight and will consider it in our future research, both qualitative and quantitative. Thank you for your feedback. |
| **Discussion** | | |
| 10 | There are some interesting elements to the discussion, particularly challenging assumptions about older age and also highlighting the importance of employment and the impact of income for working age adults. However, in this study, you are primarily assessing PA in a group of well-educated older women living in a relatively affluent region. There will be many older adults in the UK affected by poverty, ill-health, lack of digital literacy, social isolation, bereavement and so on who will have had quite different experiences over the course of the pandemic. Therefore, it is important not to over-generalise or overstate the conclusions. It needs to be clear that this is an exploratory analysis that raised interesting questions, but which cannot provide definitive answers at this stage. I would suggest pitching the conclusions more in terms of the importance of collecting data on positive mood states (as well as negative moods states and/or symptoms of psychological distress) during pandemics to enable a more holistic picture to inform policy and communication strategies. | Thank you for your thoughtful comment.  We agree that it is important to acknowledge the limitations of our study, including the selection bias of our sample, which primarily consisted of well-educated older women living in an affluent area. We also agree that our study raises interesting questions and highlights the importance of collecting data on both positive and negative mood states during pandemics to inform policy and communication strategies.  We have taken your feedback into consideration and have revised our discussion, and conclusion sections to emphasize the exploratory nature of our study and to avoid over-generalizing our findings. We appreciate your input and thank you for helping us improve the clarity and accuracy of our work. Please check the revised sections in discussion (Page 15 Line 259-279) and conclusion (Page 20-21 Line 396-413). |
