# Supplementary material for: Positive and negative emotions during the COVID-19 pandemic: A longitudinal survey study of the UK population
Source: PLoS One. 2024 Feb 7;19(2):e0297214. doi: 10.1371/journal.pone.0297214 (PMC10849398; doi:10.1371/journal.pone.0297214)
Supplement: Attachment — Submitted filename: Response to Reviewers.docx [file pone.0297214.s010.docx]

Dear Prof Marcus Tolentino Silva,

Dear Reviewers,

Thank you for giving us the opportunity to submit a revised version of our manuscript titled ‘*Positive and Negative Emotions during the COVID-19 Pandemic: A Longitudinal Survey Study of the UK Population*’ to PLOS ONE to be considered for publication as *Research Article.*

We would like to extend our sincere gratitude for your dedicated time and effort in reviewing our manuscript. Your valuable feedback has been immensely helpful in improving the quality of our work. We have taken all your comments into careful consideration and made revisions accordingly.

Notably, we have revised the rationale, strengthening its clarity and relevance. The discussion section has been thoroughly reviewed to ensure a more coherent presentation of our findings and a clear delineation of the implications of our work. Moreover, we have meticulously addressed any repetition between the discussion and conclusion, ensuring each section serves its distinct purpose.

To facilitate an easy understanding of the revisions, we have included the reviewer and editor's comments in the tables below, along with our responses and corresponding changes. All page and line numbers cited in this document correspond to the file "Manuscript."

We genuinely appreciate the time and expertise you have devoted to reviewing our work, and we are confident that the revised manuscript now meets your expectations.

Thank you once again for your invaluable feedback. We eagerly await your decision and remain grateful for your attention and valuable time.

Yours sincerely,

Lan Li

On behalf of the co-authors.

**Response to Editor**

| **Section** | **Comment** | **Response** |
| --- | --- | --- |
| Introduction | (1) Clearly state the rationale for investigating emotions during the COVID-19 pandemic and link it to the psychological underpinnings.  (2) Provide references to literature supporting the importance of emotions in predicting wellbeing and future mental health conditions. | Thank you. We have revised the introduction section and added the rationale and link to the psychological underpinnings.  Thank you. We have added the references to support this argument.  Please check the updated paragraph in page 4, line 52-63 for details. |
| Discussion | (3) Ensure that the added section in the discussion aligns with the findings presented in the study.  (4) Clearly delineate the implications of the work, addressing why the study is interesting, important, and how it can inform policy and practice.  (5) Avoid repetition between the first and last paragraph of the discussion. The first paragraph should focus on summarizing what was done and found, while the last paragraph should provide key "take-home messages." | Thank you for the helpful feedback.  We have re-written the added part to ensure the discussions align with the findings. The implication part has been refined to clearly state how the findings could inform policy and practice. Please check the updated paragraph from page 18, line 360 to page 19, line 375.  We have checked and re-written the first paragraph and conclusion to remove the repetition and clearly deliver the messages from our findings. Please find the reply to the reviewers for details. |
| Proofreading | (6) Thoroughly review the manuscript for typographical and grammatical errors. Conduct a comprehensive proofreading to ensure clarity and coherence of the writing. | Thank you for the feedback. We have conducted a thorough proofread and corrected the typo and grammatical errors. |

**Response to Reviewer #3**

| **No.** | **Comment** | **Response** |
| --- | --- | --- |
| 1 | Before this piece can be published - there is a key issue that I do not believe has been adequately addressed and this directly relates to the rationale for the study and what use understanding emotions has for healthcare professionals or policy makers in the future. In your introduction you state the aim of the work: 'This study aims to assess the change in emotions and feelings during the COVID-19 pandemic to the restrictions that were in place during the number of phases of the pandemics throughout the study period.' But you do not then go on to justify a rationale as to why you are investigating this? You state that others haven't measured emotions over a longitudinal period - but you do not address why investigating emotions is important. i.e. I assume investigating emotions is important because of links to wellbeing, predicting distress or future mental health conditions, important to know which groups are at risk and who may need support in the future. However you don't present a rationale or link to literature explaining why investigating emotions is important but make an assumption that emotions and wellbeing are tied - please cite some literature so readers can understand the psychological underpinning of where emotions sit in relation to predicting wellbeing or future mental health. This will then help your discussion. | Thank you for this key piece of feedback, and we agree that is was not adequate robust in the previous version.  We have included more language and citations, particularly in the introduction, to assert the link between emotional state, mental well-being, and resilience to disaster. Please check page 4, line 52-63 for details. |
| 2 | In your discussion you have added a section from line 365 trying to address why this research is important and what implications it might have for policy and practice - yet it doesn't seem to entirely tie in with your findings - your findings demonstrate that those who were male, younger and not employed were more likely to have negative emotions over the pandemic - hence government, local authorities, universities and voluntary organisations may also need to look at how interventions can be targeted to these groups, not only during pandemics, but these groups may have lasting effects of the pandemic on their social networks and employment. | Thank you for pointing this out. Upon re-evaluating our research, we recognize that this section might have been somewhat disjointed from the actual findings presented. We apologize for any confusion this may have caused.  In the updated version, we have revised the paragraph to better emphasize the relevance of our findings to policy and practice. Thank you for your suggestion. We have now explicitly linked the identified demographic groups who experienced more negative emotions during the pandemic to the potential need for targeted interventions. Please check the updated paragraph from page 18, line 360 to page 19, line 375. |
| **Response to Reviewer #4** | | |
| **No.** | **Comment** | **Response** |
| 1 | There is significant repetition between the first and last paragraph of the discussion and this should be reviewed. The first paragraph should focus on a brief summary of what was done and what was found. The conclusions on summarising key 'take home messages'. | Thanks for the comments. We appreciate your attention to detail and agree that some repetition has occurred between the first and last paragraphs. We apologize for this oversight.  To address this concern, we have carefully revised the first paragraph and the conclusion section to ensure clarity and eliminate unnecessary repetition. The first paragraph now provides a concise summary of our research methodology and link to the next section for key findings, while the concluding paragraph focuses on summarizing the key 'take home messages' or main conclusions drawn from the study.  Please check the updated first paragraph in discussion in page 15 and the conclusion section in page 20-21. |
| 2 | The writing is clear and of a high standard overall, but there are numerous typographical and grammatical errors throughout and thorough proof reading is required. | Thank you for the opportunity to provide a further proofread. We have carefully checked the full text and made revisions accordingly. |
